# Hepatitis E-Induced Worsening of Antiphospholipid Syndrome With Extensive Thrombosis: A Case Report

**DOI:** 10.7759/cureus.79462

**Published:** 2025-02-22

**Authors:** Abraham Karimi, Sonal Prasad, Edsel Embry, Jay Xiong, Mrinmayee Naik

**Affiliations:** 1 Medical School, Touro College of Osteopathic Medicine, Vallejo, USA; 2 Internal Medicine, St. Joseph's Medical Center, Stockton, USA

**Keywords:** antiphospholipid antibodies, antiphospholipid syndrome, aps, hepatitis e, hev, splanchnic vein thrombosis, svt

## Abstract

Antiphospholipid syndrome (APS) is an autoimmune disease that can result in a wide range of thromboembolic events. We report on a young male who presented with abdominal pain and was found to have extensive splanchnic vein thrombosis (SVT). Incidentally, he tested positive for the following: hepatitis E virus (HEV) immunoglobulin M (IgM) antibody and antiphospholipid antibodies (aPL), including anticardiolipin and anti-beta2-glycoprotein antibodies. HEV-induced worsening of APS is not a commonly documented occurrence. Cases of APS related to infection may be more prevalent than we suspect and infectious workups in patients with a history of thrombotic events could help discover possible etiologies of acute exacerbations.

## Introduction

Antiphospholipid syndrome (APS), a systemic autoimmune disease, can result in a diverse spectrum of recurrent thromboembolic events. APS can be primary or secondary to another disease state such as systemic lupus erythematosus (SLE) or infections. Certain infections such as those caused by the hepatitis A virus (HAV), hepatitis C virus (HCV), cytomegalovirus (CMV), human immunodeficiency virus (HIV), *Escherichia coli *(*E. coli*), and Leptospirosis have been associated with the development of APS [1-7].

Thromboembolic events that can result from APS include obstetrical complications such as miscarriages and arterial or venous thrombotic events. Some of the other common complications of APS include cardiac valvular disease, thrombocytopenia, and cognitive impairment [8]. Relevant to our study is the association between APS and SVT, which has been associated with certain antiphospholipid antibodies (aPL) such as immunoglobulin G (IgG) anticardiolipin [9]. APS is diagnosed based on the presence of clinical features and positive aPL. The most common aPLs used for diagnosis include the anticardiolipin antibody, anti-beta2 glycoprotein I antibody, and lupus anticoagulant. One possible explanation for infections resulting in APS is that aPL and anti-beta2-glycoprotein I antibodies can be induced by immunization with beta2-glycoprotein I-like phospholipid-binding viral and bacterial products [4]. Using clinical events and aPL titers, the American College of Rheumatology and the European Alliance of Associations for Rheumatology have developed criteria to stratify risk and management of patients with APS [10]. 

Typical management for an acute thrombosis with suspected APS includes low molecular weight heparin followed by warfarin. Long-term anticoagulation can also be achieved with warfarin as it has been demonstrated to be effective in preventing recurrent thrombosis in APS patients [11, 12]. In this study, we present a young male with abdominal pain who was found to have extensive splanchnic vein thrombosis (SVT). Incidentally, he was positive for HEV IgM antibodies.

## Case presentation

A healthy 22-year-old male with no known past medical history presented with worsening abdominal pain. On admission, abdominal pain was localized to the gastric and periumbilical regions. Associated symptoms included nausea, vomiting, and absence of bowel movement in the past three days. His vital signs demonstrated tachycardia at 108 beats per minute but were otherwise normal on admission. Notable lab findings include an elevated white blood cell count of 13.2 thousand/uL (normal range is 4.0-10.0 thousand/uL), mild hypokalemia of 3.4 mmol/L (normal range is 3.5-5.1 mmol/L), an elevated alkaline phosphatase of 214 units/L (normal range is 40-150 units/L). Table 1 summarizes the patient’s vitals and laboratory results.

**Table 1:** Vitals and labs

Vitals/labs	Range	Reference ranges
Temperature	36.8 deg C	36-37.5 deg C
Heart rate	108	60-99
Respiratory rate	14	13-20
Blood pressure	130/80	91-139 / 51-89
SpO2	98% on room air	>92%
White blood cells	13.2 thousand/uL	4.0-10.0 thousand/uL
Hemoglobin	13.1 gm/dL	13.6-17.0 gm/dL
Hematocrit	39.00%	39-49%
Platelets	219 thousand/uL	150-400 thousand/uL
Sodium	140 mmol/L	136-145 mmol/L
Potassium	3.4 mmol/L	3.5-5.1 mmol/L
Chloride	102 mmol/L	98-107 mmol/L
CO2	25.0 mmol/L	22-29 mmol/L
Anion gap	13	5-14 mmol/L
Glucose	124 mg/dL	70-105 mg/dL
Blood urea nitrogen	12.3 mg/dL	8.4-25.7 mg/dL
Creatinine	0.8 mg/dL	0.7-1.3 mg/dL
eGFRcr	>90 mL/min	>90 mL/min
Calcium	9.2 mg/dL	8.4-10.5 mg/dL
Total bilirubin	0.7 mg/dl	0.1-1.2 mg/dL
Alanine aminotransferase	54 Unit/L	0-55 Units/L
Aspartate aminotransferase	20 Unit/L	5-34 Unit/L
Alkaline phosphatase	214 Unit/L	40-150 Units/L
Lipase	5 Unit/L	8-78 Units/L
Lactic acid	1.4 mmol/L	0.5-2.0 mmol/L
Hepatitis B surface antibody	0.55 IU/mL	< 8.00 IU/mL
Hepatitis B surface antigen	Non-reactive	
Hepatitis A antibody, IgM	Negative	
Hepatitis A antibodies (total)	Positive	
Hepatitis B core antibody (total)	Non-reactive	
Hepatitis C antibody	Non-reactive	
HEV IgM	Detected	

**Figure 1:**
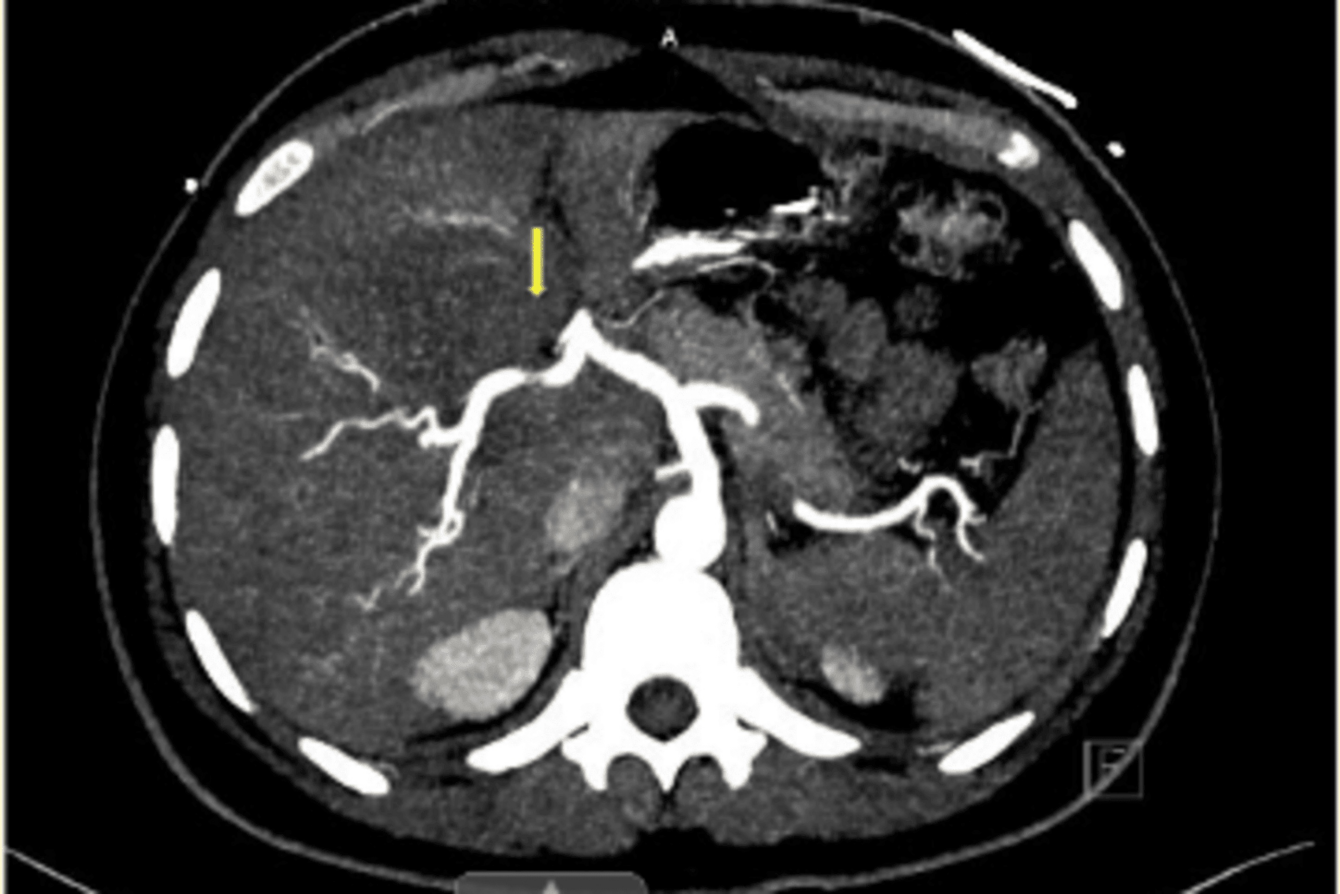
CT angiography of abdomen/pelvis showing thrombosis of the portal vein (yellow arrow)

**Figure 2:**
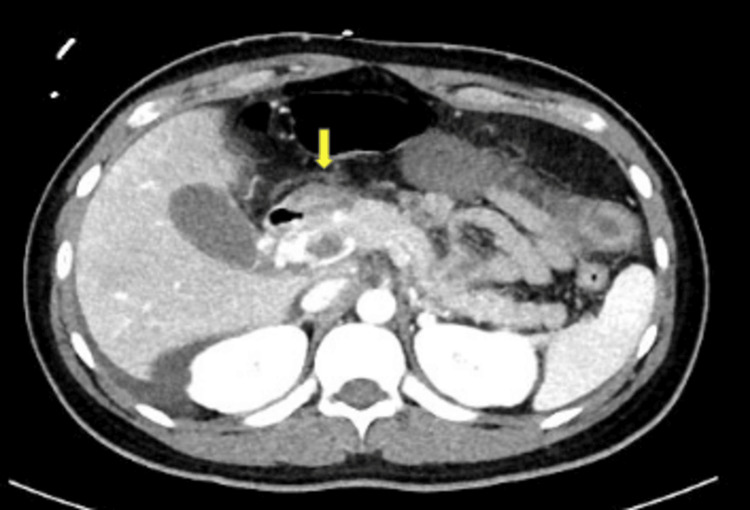
CT angiography of abdomen/pelvis showing thrombosis of the portal vein (yellow arrow)

**Figure 3:**
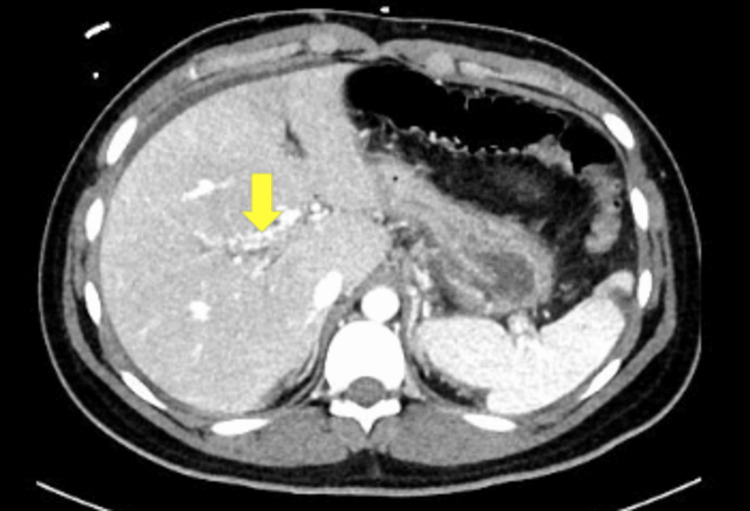
Cavernous transformation of the portal vein (yellow arrow)

**Figure 4:**
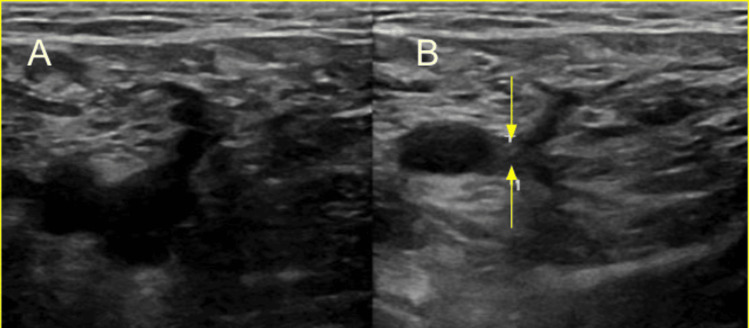
Panel A (left) showing left common femoral vein. Panel B (right) showing right common femoral noncompressible vein indicating thrombus (yellow arrows)

**Figure 5:**
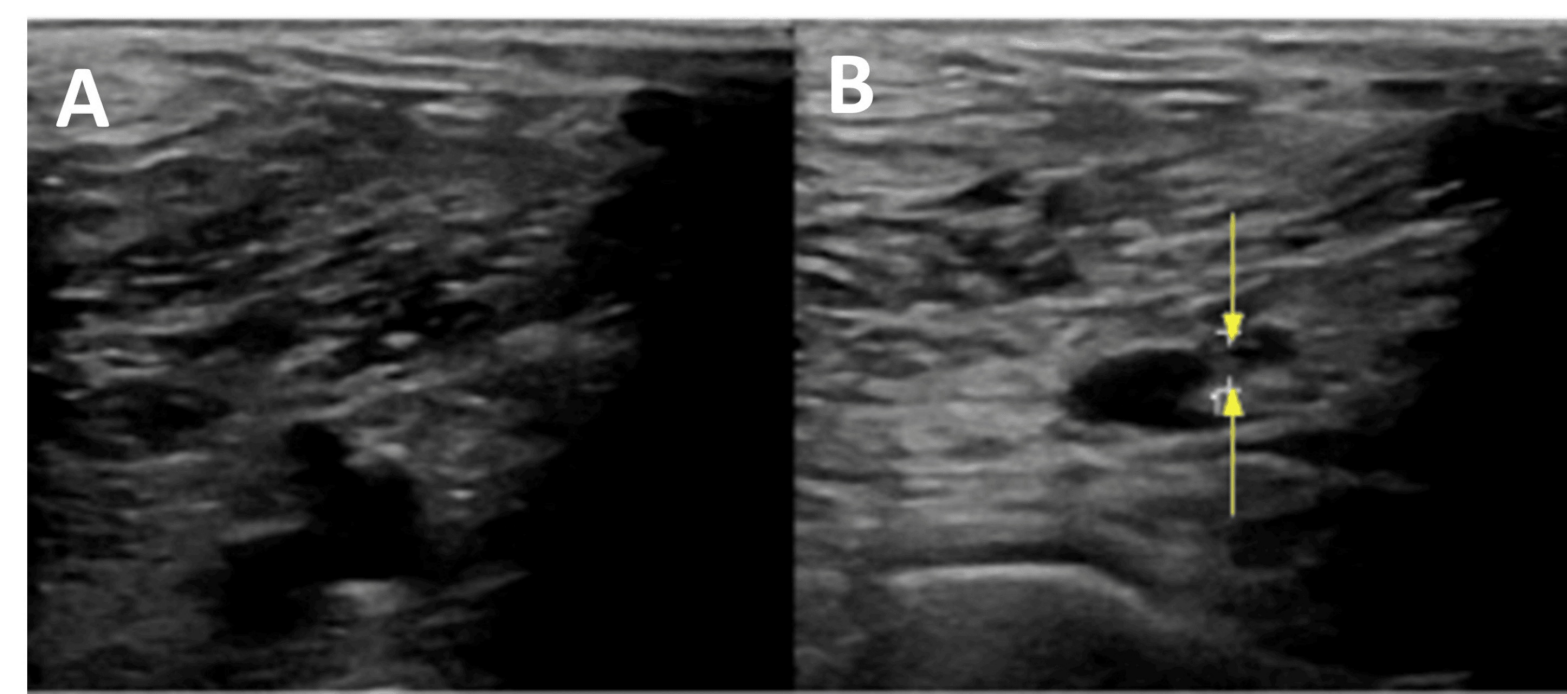
Panel A (left) showing left popliteal vein. Panel B (right) showing right popliteal noncompressible vein indicating thrombus (yellow arrows)

Lactic acid was normal at this time but was measured after the patient completed a lactated ringer’s bolus. The positive hepatitis A total antibody test along with a negative hepatitis A IgM antibody test is consistent with a recovered hepatitis A infection or response from a vaccination. The only notable finding from the hepatitis panel was a detected HEV IgM. His urinalysis showed a sterile pyuria. The patient was started on intravenous (IV) ceftriaxone 1000mg daily and IV metronidazole 500mg every eight hours. Computed tomography (CT) angiography of the abdomen and pelvis with contrast showed thrombosis of the portal vein (Figures 1-2). The CT also demonstrated cavernous transformation where multiple serpiginous tiny vessels extend along the portal vein (Figure 3). The cavernous transformation indicates that there was likely a chronic pathology involved. Duplex ultrasound of the bilateral lower extremities showed partially occlusive noncompressible deep vein thrombosis in the common femoral and popliteal veins which sided towards a more acute pathology (Figures 4-5). 

The patient was started on a heparin drip. He was managed with bowel rest and a proton pump inhibitor. Diagnosis of APS was made as anticardiolipin antibody IgM was slightly positive at 16 micrograms of IgM antibody (normal range is ≤ 12) and anti-beta2-glycoprotein IgG was positive at 58 micrograms of IgG antibody (normal range is ≤ 20) in the setting of venous thrombosis. 

**Figure 6:**
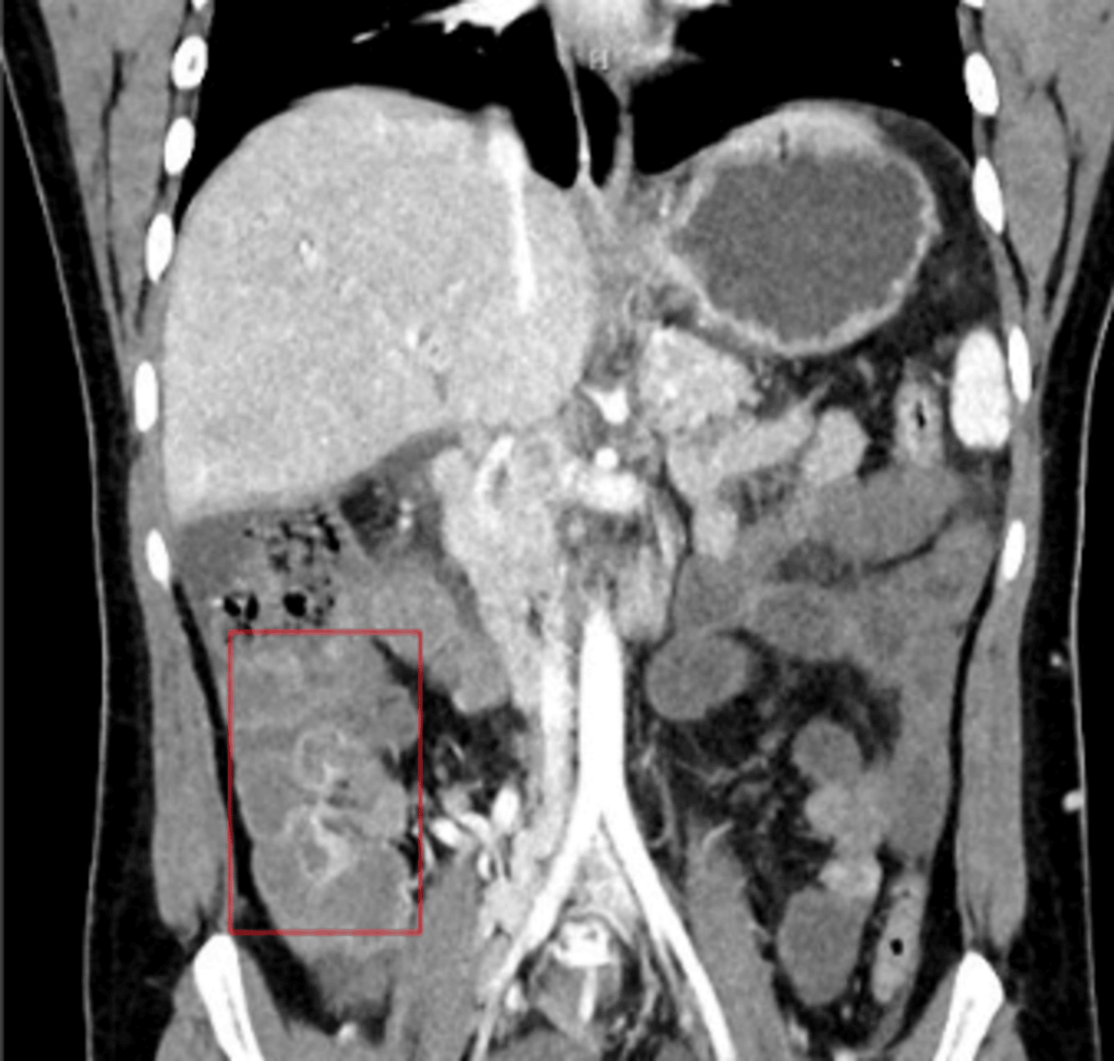
CT abdomen/pelvis showing increased small bowel wall thickening and hyperenhancement of loops of bowel (red box)

Janus kinase 2 and paroxysmal nocturnal hemoglobinuria screens were negative. Complete thrombophilia workup showed low antithrombin of 74% (normal range is 80-120%). The decision was made to transition to warfarin, however, after two doses of warfarin. The INR was supratherapeutic at 4.5 (normal range is 2.0 - 3.0) and we chose to administer a heparin drip until better control was obtained. Six days after admission, the patient started complaining of worsening abdominal pain. A repeat CT of the abdomen and pelvis with and without IV contrast showed increasing small bowel wall thickening and hyperenhancement of loops of the bowel concerning for developing venous mesenteric ischemia (Figure 6).

Lactic acid increased to 2.5 mmol/L (normal range is <2 mmol/L), WBC increased to 33.3 thousand/uL (normal range is 4-11 thousand/uL), and INR was 6.7 (normal range is 2.0-3.0). The decision was made to transfer the patient to a tertiary center for an interventional thrombectomy.

## Discussion

The pathogenesis of APS is complex and multiple factors can play a role in this disease. Pertinent to this study is the relationship between APS and SVT. SVT that coincides with APS has been associated with the aPL IgG anticardiolipin [9]. In this study, the patient's IgM anticardiolipin was slightly positive at 16 and their anti-beta2-glycoprotein IgG was positive at 58. APS is diagnosed based on the presence of clinical features and positive aPL.

This patient’s presentation is complicated by the fact that cavernous transformation of the portal vein indicates chronic changes. However, his clinical presentation and HEV IgM could signal that the viral infection induced a worsening of their hypercoagulable state, indicated by small bowel wall thickening, hyperenhancement of loops of the bowel, and noncompressible popliteal and common femoral thromboses. This acute worsening could be due to systemic inflammation from the hepatitis leading to hypercoagulability. Another possibility is that the production of antiphospholipid antibodies was induced by the binding of viral products [4].

There are several case reports and studies that have described an association between infection and an increased risk of developing APS, with substantially more literature focused on HCV and HIV [1-7]. An association with HEV does not appear to be common. In our research, we were unable to find similar cases of HEV-inducing symptoms of APS. However, there are documented cases of other types of hepatitis causing similar symptoms [2]. It is possible that the patient had another underlying pathology causing their chronic changes that went undetected during our workup and that their HEV infection contributed to an acute worsening of the thrombotic process. The existing literature does not clearly explain the specific mechanism by which APS occurs in these infections.

## Conclusions

HEV is a common cause of acute viral hepatitis worldwide. HEV IgM is typically detected within two to four weeks after infection and can persist for up to two months. There is a high likelihood that HEV could be contributing to more occurrences of worsning APS and thrombotic events than we are aware of. However, the exact mechanism remains unknown.

In a patient with no history of APS, an infectious workup can help discover possible etiologies. Advances in this area should focus on identifying specific infections that can lead to or acutely worsen APS, as well as understanding the underlying pathogenesis. Further research in figuring out specific aPL associated with each infectious cause would also be beneficial in discovering the pathophysiology of infection-related APS. This would also help distinguish between APS, which is caused by infection, and another pathology.
